# Silencing of NLRP3 Sensitizes Chemoresistant Ovarian Cancer Cells to Cisplatin

**DOI:** 10.1155/2023/7700673

**Published:** 2023-06-02

**Authors:** Weijia Li, Xibo Zhao, Rujian Zhang, Jiabin Xie, Guangmei Zhang

**Affiliations:** ^1^Department of Gynecology, Harbin Medical University, Harbin, 150081 Heilongjiang, China; ^2^Department of Gynecology, The First Affiliated Hospital of Harbin Medical University, Harbin, 150081 Heilongjiang, China; ^3^Department of Gynecology, Southern Medical University Affiliated Maternal and Child Health Hospital of Foshan, Foshan, 528000 Guangdong, China

## Abstract

**Background:**

Ovarian cancer is a fatal gynecological malignancy. The resistance to chemotherapy in ovarian cancer treatment has been a thorny issue. This study is aimed at probing the molecular mechanism of cisplatin (DDP) resistance in ovarian cancer.

**Methods:**

Bioinformatics analysis was conducted to examine the role of Nod-like receptor protein 3 (NLRP3) in ovarian cancer. The NLRP3 level in DDP-resistant ovarian cancer tumors and cell lines (SKOV3/DDP and A2780/DDP) was evaluated by applying immunohistochemical staining, western blot, and qRT-PCR. Cell transfection was conducted to regulate the NLRP3 level. Cell abilities to proliferate, migrate, invade, and apoptosis were measured employing colony formation, CCK-8, wound healing, transwell, and TUNEL assays, respectively. Cell cycle analysis was completed via flow cytometry. Corresponding protein expression was measured by western blot.

**Results:**

NLRP3 was overexpressed in ovarian cancer, correlated with poor survival, and upregulated in DDP-resistant ovarian cancer tumors and cells. NLRP3 silencing exerted antiproliferative, antimigrative, anti-invasive, and proapoptotic effects in A2780/DDP and SKOV3/DDP cells. Additionally, NLRP3 silencing inactivated NLRPL3 inflammasome and blocked epithelial-mesenchymal transition via enhancing E-cadherin and lowering vimentin, N-cadherin, and fibronectin.

**Conclusion:**

NLRP3 was overexpressed in DDP-resistant ovarian cancer. NLRP3 knockdown hindered the malignant process of DDP-resistant ovarian cancer cells, providing a potential target for DPP-based ovarian cancer chemotherapy.

## 1. Introduction

Ovarian cancer, well-prevalent gynecological oncology, is one of the main contributors to deaths associated with cancer in females worldwide [1, 2]. It is estimated that the new ovarian cancer cases may rise to 371000, and the number of deaths will be 254000 by 2035 [3]. Early ovarian cancer is not easily diagnosed due to ineffective screening means, [4] resulting in its development to an advanced stage when diagnosed. Surgical resection combined with cisplatin- (DDP-) based chemotherapy is the mainstay of ovarian cancer treatment. Nevertheless, 70%-90% of the patients relapse, and most develop resistance to the DPP challenge, which takes the main responsibility for the treatment failure and the poor 5-year survival rate below 40% [5, 6]. Thus, clarifying the potential mechanism of chemoresistance and developing effective strategies for reducing DDP resistance in ovarian cancer are quite essential.

Nod-like receptor protein 3 (NLRP3) inflammasome is a complex consisting of NLRP3, the adaptor apoptosis-associated speck-like protein (ASC), and the effector pro-caspase1. NLRP3 inflammasome activation facilitates the formation of active caspase-1 and contributes to the maturity and production of interleukin (IL)-18 and IL-1*β*, eventually leading to inflammation [7–9]. As a crucial constituent of the innate immune system, the NLRP3 inflammasome frequently responds to cellular damage and microbial infection [10]. Recently, emerging evidence revealed that NLRP3 inflammasome was dysregulated during tumor development. For example, NLRP3 was overexpressed in colon cancer cells and linked to the poor survival of patients [11, 12]; NLRP3 inflammasome was activated in gastric cancer, which was beneficial in promoting tumorigenesis [13]. On the contrary, NLRP3 inflammasome was downregulated in human hepatocellular carcinoma, and the absent NLRP3 was linked to advanced stages [14]. Thus, attributed to the dual role in different types of malignant tumor, NLRP3 inflammasome was regarded as a double-edged sword in tumorigenesis [15]. Of note, NLRP3 was reported to have an aberrantly high expression in ovarian cancer, and NLRP3 inflammasome participated in the development of ovarian cancer [16, 17]. However, a knowledge gap exists concerning the impacts of NLRP3 inflammasome on ovarian cancer with DPP resistance.

Here, we aimed to assess the expression level of NLRP3 in ovarian cancer with DDP resistance, clarify its potential regulatory role, and offer new therapeutic strategies for the advancement of chemoresistance in ovarian cancer. The graphical flow chart has been illustrated in Scheme 1.

## 2. Methods and Materials

### 2.1. Bioinformatics Analysis

Gene Expression Omnibus (GEO; https://www.ncbi.nlm.nih.gov/geo/) database includes experimental data for genomic DNA and proteins and data from single- and dual-channel determination of mRNA expression. Five expression profiling datasets (GSE26712, GSE53963, GSE51088, GSE66957, and GSE135886) were obtained from the GEO database. The expression data of NLRP3 in ovarian cancer were analyzed using GEO2R online. The figure construction was carried out, adopting R package software 4.0.3. A total of 381 ovarian cancer cases and 71 normal cases were downloaded from The Cancer Genome Atlas (TCGA) database (http://www.cancer.gov/tcga). The expression of NLRP3 in ovarian cancer tumor tissues and normal tissues was analyzed according to the downloaded data. The clinical profile of 381 ovarian cancer patients was obtained from the TCGA database, and the survival analysis was conducted using the Kaplan-Meier method. The patients with missing clinical data about tumor stages were excluded from this study. A total of 377 samples (23 at stage II, 296 at stage III, and 58 at stage IV) were examined for NLRP3 level at different tumor stages. Moreover, a single-variable Cox proportional risk regression assay was conducted to explore the risk factors that were remarkably correlated with overall survival in TCGA ovarian cancer dataset. In addition, Tumor Immunization Estimation Resource (TIMER; http://timer.cistrome.org), a comprehensive database to assess the relationship between immune infiltrating cells and overall survival of cancers, was applied to estimate the infiltration of CD8^+^ T cells, CD4^+^ T cells, B cells, neutrophils, macrophage, and myeloid dendritic cells related to NLRP3 level in ovarian cancer patients.

### 2.2. Tissue Samples

A total of 36 ovarian cancer specimens were collected from patients receiving oophorectomies from 2022.1 to 2022.10 at Foshan Women and Children Hospital. Among these specimens, 18 were harvested from DDP-resistant patients suffering from recurrent or persistent disease within 6 months following DDP-based chemotherapy. The other was collected from DDP-sensitive (nonresistant) patients without recurrence or with recurrence beyond 6 months. The patient's clinical information is listed in Table 1. The study was approved by the Ethics Committees of Foshan Women and Children Hospital (Approval number: FSFY-MEC-2021-145), and written consent was acquired from all patients.

### 2.3. Immunohistochemical Staining

The tissue was fixed in 4% paraformaldehyde for 24 h, embedded in paraffin, and sliced to a thickness of 5 *μ*m. Subsequently, the slices were deparaffinized and rehydrated, followed by heating in 0.01 M citric buffer for 15 min. Before probing overnight at 4°C with anti-NLRP3 antibody (ab263899, Abcam), the slices were impeded in 3% H_2_O_2_ for 20 min and then with 5% normal serum for 30 min. Thereafter, the slices were probed with horseradish peroxidase- (HRP-) conjugated secondary antibody (ab6721, Abcam) for 30 min, diaminobenzidine (DAB) stained (ZSGB-BIO, Beijing, China) for 5 min followed by hematoxylin counterstaining for 30 s. Eventually, the images were obtained utilizing a light microscope (Olympus, Tokyo, Japan).

### 2.4. Quantitative Real-Time Polymerase Chain Reaction (qRT-PCR)

RNA content was exacted from the tumor tissues adopting Trizol (Thermo Fisher Scientific, CA, USA). The concentration and purity of the total RNA were checked using NanoDrop 3000 (Thermo Fisher Scientific Inc., Waltham, MA, USA). The RNA content was reversely transcribed into complementary DNA (cDNA) adopting a cDNA synthesis kit (Thermo Fisher Scientific Inc.), followed by qRT-PCR analysis utilizing SYBR Green Master Mix (Takara, Shiga, Japan). The primers of PCR are listed in Table 2. 2^−△△Ct^ method was utilized for calculating gene expressions using *β*-actin as an internal reference.

### 2.5. Western Blot

RIPA lysis buffer (Bolingkewei, Beijing, China) was applied to homogenize the tissues. The whole protein was extracted and quantified using a bicinchoninic acid (BCA) protein assay kit (Pierce, Rockford, IL, USA). The protein (35 *μ*g/lane) was isolated through 12% SDS-PAGE gels, followed by transferring onto polyvinylidene difluoride (PVDF) membranes (Millipore, MA, USA). After 1 h incubation with 5% defatted milk, membranes were probed at 4°C overnight against the following antibodies: NLRP3 (ab263899, Abcam), IL-18 (ab243091, Abcam), cleaved caspase-1 (orb126550, Biorbyt), IL-1*β* (ab216995, Abcam), N-cadherin (ab76011, Abcam), E-cadherin (ab40772, Abcam), fibronectin (ab2413, Abcam), vimentin (ab92547, Abcam), and *β*-actin (ab8226, Abcam). On the following day, membranes were exposed to goat anti-mouse (ab6789, Abcam) or goat anti-rabbit (ab6721, Abcam) HRP-conjugated secondary antibodies for 2 h at room temperature. Eventually, the signals were developed with an enhanced chemiluminescence system (Millipore, USA), followed by quantification with ImageJ software (NIH, Bethesda, Maryland, USA).

### 2.6. Cell Culture and Treatment

A2780 and SKOV3, two human ovarian cancer cell lines, were provided by BeNa Culture Collection (Beijing, China). SKOV3 cells were cultivated in McCoy's 5a medium. In contrast, A2780 cells were cultivated in RPMI-1640 medium (Hyclone, Logan, UT, USA) in a 5% CO_2_ environmental incubator at 37°C, supplemented with 10% fetal bovine serum (FBS; Gibco, USA) with 0.1 mg/mL streptomycin and 100 U/mL penicillin (Gibco, USA). DDP-resistant SKOV3/DDP and A2780/DDP cells were constructed as previously reported [18]. 0.5 *μ*g/L of DDP was added for SKOV3/DDP and A2780/DDP cells to retain the resistance to DDP.

### 2.7. Cell Transfection

Short hairpin RNA (shRNA) targeting NLRP3 (sh-NLRP3) was designed and synthesized by RiboBio (Guangzhou, China). Cells transfection using sh-NLRP3 and sh-vector (negative control) was implemented utilizing a Lipofectamine 3000 Transfection reagent (Invitrogen, CA, USA) strictly in line with its guidelines. Subsequent experiments were carried out 48 h posttransfection.

### 2.8. Cell Counting Kit-8 (CCK-8) Assay

5 × 10^3^ cells were cultivated into 96-well plates. CCK-8 reagent (Dojindo Molecular Technologies, Gaithersburg, MD) was added to the plates at indicated times (20 l), and the plates were incubated for 2 hours. The absorbance was tested at 450 nm with the help of a microplate reader (Bio-Rad Laboratories, Hercules, CA).

### 2.9. Colony Formation Assay

2 × 10^3^ cells were plated in 6-well plates and then cultivated for ten days. During this period, the medium was replaced every three days. 4% paraformaldehyde was employed to immobilize the colonies for 20 min. Eventually, cells underwent staining with 0.1% crystal violet for 30 min, followed by observation and counting.

### 2.10. Wound-Healing Assay

5 × 10^4^ cells were cultivated into 6-well plates in a 5% CO_2_ incubator at 37°C. Upon achieving 100% confluence, a sterile pipette tip was adopted to create liner scratches. PBS washed the plates carefully to discard the floating cells. 24 h later, the healing of the scratches was observed and photographed employing a light microscope (Olympus, Tokyo, Japan).

### 2.11. Transwell Assay

A 24-well insert transwell chamber (Millipore, MA, USA) was coated with 200 mg/ml Matrigel (BD Biosciences, NY, USA). 5 × 10^4^ cells were resuspended in serum-free medium and placed into the upper transwell chamber, while 500 *μ*l medium with 10% FBS was put into the lower chamber. 24 h later, after wiping out the noninvasive cells, the cells got a fixation with 4% paraformaldehyde, followed by 0.5% crystal violet staining. Images of invaded cells were photographed by a light microscope (Olympus, Tokyo, Japan).

### 2.12. Terminal Deoxynucleotidyl Transferase-Mediated dUTP Nick End Labeling (TUNEL) Staining

4% paraformaldehyde was utilized to fix cells. 20 min later, cells underwent permeabilization using 0.1% Triton X-100 for 30 minutes and inactivated endogenous peroxidase by 0.3% H2O2 for 20 minutes. Afterward, the TUNEL mixture solution (Beyotime and Biotechnology, Shanghai, China) was added, and the cells were incubated for 1 h at 37°C in the darkness. 4′, 6-diamidino-2-phenylindole (DAPI) solution was added to stain cells. The images were captured adopting an Olympus IX70 inverted microscopy (Olympus, Tokyo, Japan).

### 2.13. Flow Cytometry Analysis

Cells were washed with precooled PBS at 4°C, followed by fixation in 70% ethanol overnight at 4°C. After that, cells were stained with 50 *μ*g/mL propidium iodide (PI) containing RNase (Beyotime and Biotechnology, Shanghai, China) for 30 min in the darkness. The cell cycle was measured adopting flow cytometry (FACSCalibur, Becton-Dickinson) and analyzed using FlowJo software (Leonard Herzenberg, Stanford University, USA).

### 2.14. Statistical Analysis

All data analyzed by GraphPad Prism 8.0 (GraphPad, CA, USA) were presented as mean ± standard deviation. Group comparisons were evaluated by adopting a one-way analysis of variance (ANOVA) with Tukey's post hoc test. *p* < 0.05 meant statistically significant.

## 3. Results

### 3.1. NLRP3 Is Overexpressed in Ovarian Cancer and Linked to Poor Prognosis

**Figure 1:**
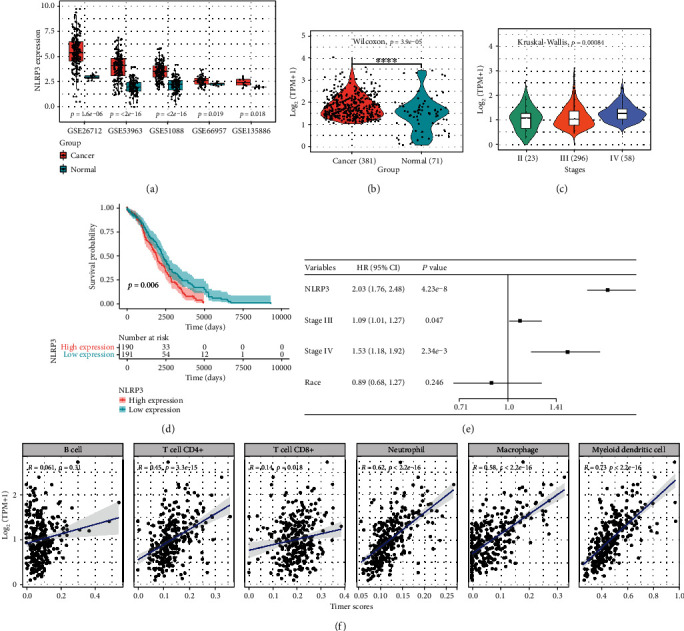
NLRP3 is overexpressed in ovarian cancer and linked to poor prognosis. (a) The gene expression data for ovarian cancer patients with NLRP3 expression information (GSE26712, GSE53963, GSE51088, GSE66957, and GSE135886) were obtained from the NCBI GEO database. (b) The expression profile of NLRP3 in ovarian cancer from TCGA database. (c) NLRP3 level in different clinical stages of patients with ovarian cancer. (d) The survival rate analysis of NLRP3 level and ovarian cancer patients. (e) Cox regression analysis of NLRP3 in ovarian cancer. (f) TIMER database was adopted to assess the association between NLRP3 level and immune infiltrates' abundances in ovarian cancer.

Based on the indicated role of NLRP3 in malignant diseases, we analyzed the expression profile of NLRP3 in ovarian cancer depending on GEO and TCGA databases. As shown in Figure 1(a), the public data from the GEO data portal revealed that NLRP3 expression was upregulated in tumor tissues of ovarian cancer, compared to the normal, which was concordant with the findings from the TCGA database (Figure 1(b)). Meanwhile, the expression level of NLRP3 was increasing from stage II to stage IV of ovarian cancer patients (Figure 1(c)), suggesting that NLRP3 was positively linked to the deterioration of ovarian cancer. The survival assay followed by Cox regression analysis revealed that patients with a higher level of NLRP3 had a poorer survival probability, and NLRP3 might act as an independent prognostic gene in ovarian cancer (Figures 1(d) and 1(e)). Finally, immune infiltration analysis using TIMER2 found that NLRP3 expression levels were significantly positively linked to the proportions of T cells, neutrophils, macrophages, and myeloid dendritic cells in ovarian cancer tissues (Figure 1(f)).

### 3.2. NLRP3 Is Upregulated in DPP-Resistant Ovarian Cancer

**Figure 2:**
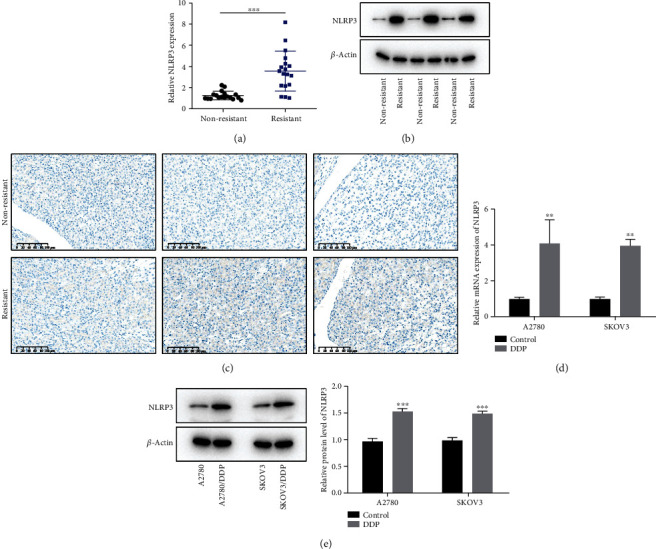
NLRP3 is upregulated in DPP-resistant ovarian cancer. (a) The tumor tissues from ovarian cancer patients with DDP resistance or not were collected, and the mRNA level of NLRP3 in tumor tissues was assessed by qRT-PCR. (b) The NLRP3 expression in tumor tissues was assessed using a western blot. (c) The NLRP3 expression in tumor tissues was observed using immunohistochemical staining. (d) The NLRP3 level in A2780 and SKOV3 and the DDP-resistant ovarian cancer cells was assessed, adopting qRT-PCR. (e) The NLRP3 expression in A2780 and SKOV3 and the DDP-resistant ovarian cancer cells was assessed adopting a western blot. ^∗∗^*p* < 0.01, and ^∗∗∗^*p* < 0.001.

To understand the role of NLRP3 in DDP-resistant ovarian cancer, we first detected NLRP3 levels in patients with DPP resistance. As shown in Figure 2(a), the NLRP3 level in DDP-resistant tumor tissues was greatly higher than that in nonresistant tissues. Subsequently, three unpaired tumor tissues from DDP-resistant and non-resistant patients were randomly measured, employing immunohistochemical staining and western blot. It was observable that the NLRP3 level was remarkably upregulated in resistant tissues (Figures 2(b) and 2(c)). Furthermore, we evaluated the NLRP3 level in ovarian cancer cell lines (A2780 and SKOV3) and their DDP-resistant forms and found that NLRP3 was upregulated in A2780/DDP and SKOV3/DDP cells (Figures 2(d) and 2(e)).

### 3.3. Silencing of NLRP3 Retard Cell Proliferation and Cell Cycle Progression in DDP-Resistant Ovarian Cancer Cells

**Figure 3:**
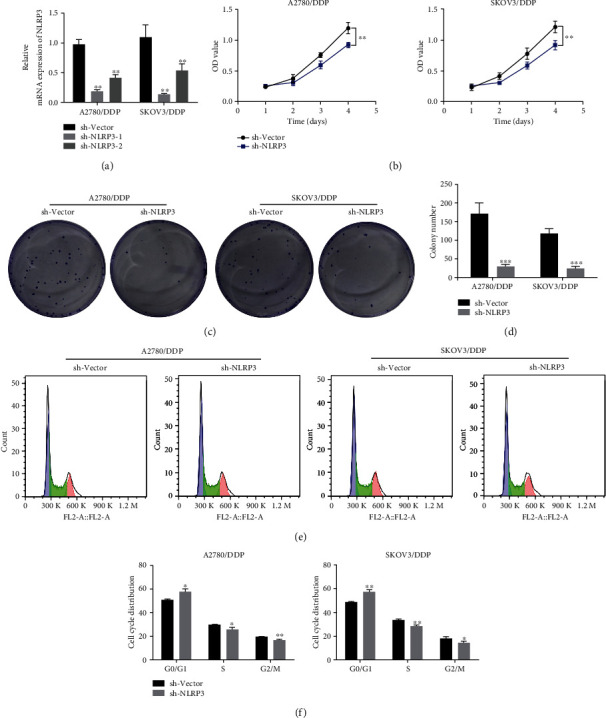
Silencing of NLRP3 suppressed cell proliferation and cell cycle progression in DDP-resistant ovarian cancer cells. (a) Both A2780/DDP and SKOV3/DDP cells received transfection using sh-NLRP3-1 and sh-NLRP3 to knock down NLRP3, and the mRNA level of NLRP3 was detected using qRT-PCR. (b) The cell viability was assessed, adopting CCK-8 assay. (c, d) Colony formation assay was conducted to examine cell proliferation. (e, f) Cell cycle was assessed using flow cytometry. ^∗^*p* < 0.05, ^∗∗^*p* < 0.01, and ^∗∗∗^*p* < 0.001.

Next, a series of cellular biological activities were measured to assess the regulation of NLRP3 in DDP-resistant ovarian cancer. A2780/DDP and SKOV3/DDP cells were transfected with sh-NLRP3 to knock down NLRP3. Attributed to a relatively high transfection efficacy, sh-NLRP3-1 was selected in the following experiments (Figure 3(a)). The findings, as shown in Figure 3(b), revealed that the cell viability in the sh-NLRP3 group was significantly reduced in A2780/DDP and SKOV3/DDP cells compared to the sh-vector group. Meanwhile, the colonies were also lessened following NLRP3 knockdown (Figures 3(c) and 3(d)). In addition, flow cytometry analysis exhibited an elevated cell cycle arrest in G0/G1 phase, accompanied by a reduced cell proportion in the S phase after NLRP3 knockdown in both A2780/DDP and SKOV3/DDP cells (Figures 3(e) and 3(f)), suggesting that the cell cycle progression was blocked by NLRP3 knockdown in DPP-resistant ovarian cancer cells.

### 3.4. Silencing of NLRP3 Represses Cell Invasion and Migration While Promoting Apoptosis in DDP-Resistant Ovarian Cancer Cells

**Figure 4:**
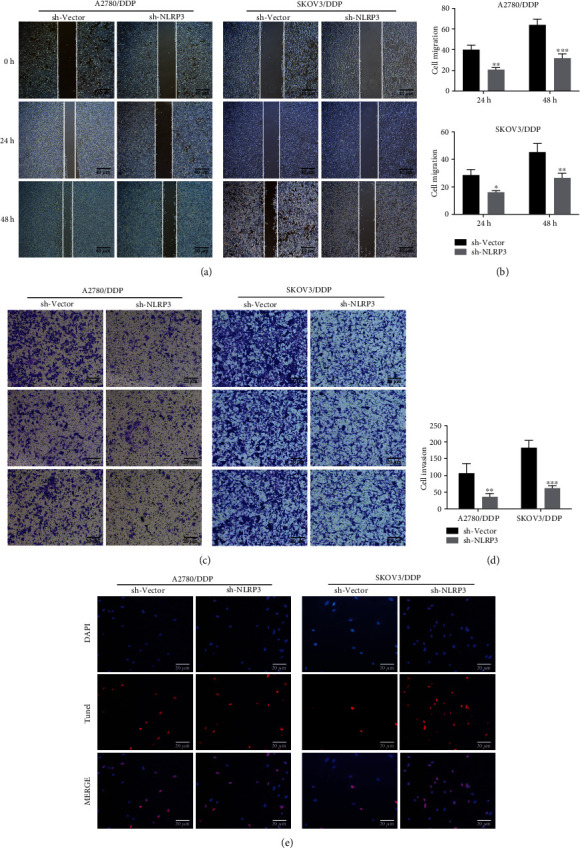
Silencing of NLRP3 repressed cell invasion and migration while facilitated apoptosis in DDP-resistant ovarian cancer cells. (a, b) Cell ability to migrate was assessed, adopting a wound-healing assay. (c, d) Cell ability to invade was assessed, adopting transwell assay. (e) TUNEL assay was carried out to detect cell apoptosis and apoptosis. ^∗^*p* < 0.05, ^∗∗^*p* < 0.01, and ^∗∗∗^*p* < 0.001.

Subsequently, a series of cellular behaviors were examined to assess cell migration, invasiveness, and apoptosis changes after NLRP3 silencing. As presented in Figures 4(a) and 4(b), the healing of the scratch was hindered in the sh-NLRP3 group compared to the sh-vector group in both SKOV3/DPP cells and A2780/DDP cells, suggesting that the migration ability of these DDP-resistant cells was weakened upon following NLRP3 knockdown. Meanwhile, the less invasive cells observed in the sh-NLRP3 group in Figure 4(c) indicate that NLRP3 silencing weakened the invasive ability of DDP-resistant ovarian cancer cells. Afterward, Figure 4(e) revealed that the silencing of NLRP3 caused a remarkable elevation of TUNEL-positive cells in SKOVE/DDP cells but not A2780/DDP cells.

### 3.5. Silencing of NLRP3 Inactivates NLRP3 Inflammasome in DDP-Resistant Ovarian Cancer Cells

**Figure 5:**
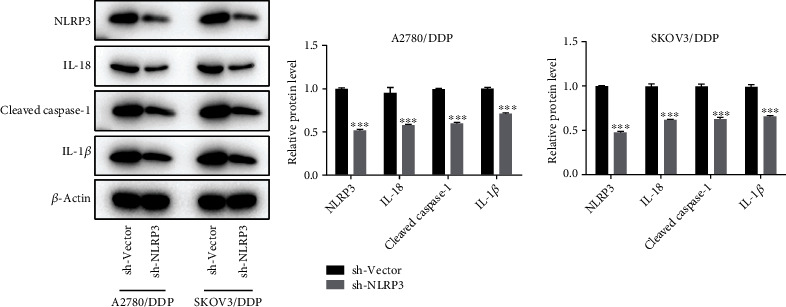
Silencing of NLRP3 inactivated NLRP3 inflammasome in DDP-resistant ovarian cancer cells. The protein expression of NLRP3, IL-18, IL-1*β*, and cleaved caspase-1 was assessed, adopting a western blot. ^∗∗∗^*p* < 0.001.

As NLRP3 is a crucial component of the NLRP3 inflammasome, we examined the impact of NLRP3 silencing on the NLRP3 inflammasome.  Figure 5 shows that the expression level of NLRP3, IL-18, IL-1*β*, and cleaved caspase-1 was significantly reduced following NLRP3 knockdown in both SKOVE/DDP cells and A2780/DDP cells, demonstrating that silencing of NLRP3 inactivated NLRP3 inflammasome in DDP-resistant ovarian cancer cells.

### 3.6. Silencing of NLRP3 Suppresses Epithelial-Mesenchymal Transition (EMT) in DDP-Resistant Ovarian Cancer Cells

**Figure 6:**
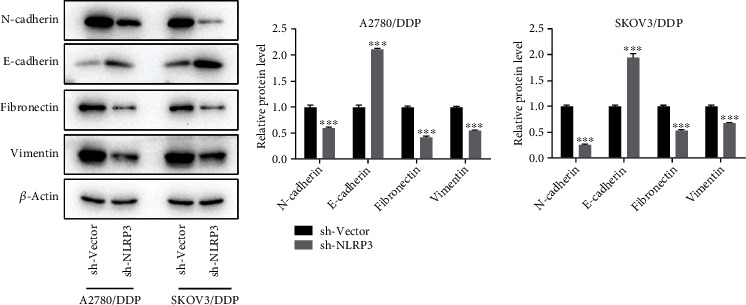
Silencing of NLRP3 suppressed EMT in DDP-resistant ovarian cancer cells. The protein expression of vimentin, fibronectin, N-cadherin, and E-cadherin was assessed, adopting a western blot. ^∗∗∗^*p* < 0.001.

EMT is an early event of tumor invasion and metastasis [19]; hence, we examined the impacts of NLRP3 knockdown on EMT markers. As exhibited in Figure 6, the epithelial marker E-cadherin was significantly upregulated following NLRP3 silencing, but the expression level of N-cadherin, fibronectin, and vimentin, the mesenchymal markers, were markedly lowered following NLRP3 knockdown, indicating that NLRP3 knockdown reversed EMT in DDP-resistant ovarian cancer cells.

## 4. Discussion

Ovarian cancer is a fatally gynecological malignant tumor. Attributed to drug resistance, it is hard to completely cure, leading to recurrences, metastasis, and poor survival rate of ovarian cancer [20]. Hence, it is essential to elucidate the drug-resistance mechanism and to prevent drug resistance in ovarian cancer. Here, it is the first time to be verified that NLRP3 serves a vital role in DDP-based chemoresistance of ovarian cancer. The findings revealed that NLRP3 was overexpressed in response to DDP-resistant ovarian cancer. NLRP3 knockdown could effectively block cell proliferation, invasion, and EMT, while it promotes apoptosis in ovarian cancer cells with DDP resistance. Collectively, the data revealed that NLRP3 knockdown and the inactivation of NLRP3 inflammasome might weaken the malignant phenotype of ovarian cancer with DDP resistance and regulate the chemoresistance of ovarian cancer cells to DDP-based therapy.

The involvement of NLRP3 inflammasome in tumor initiation and development of different types of cancer has been widely addressed, especially its dual role in cancers as aforementioned. As drug resistance strictly limits the therapeutic efficacy of chemotherapy and seriously harms patients' health and life, increasing attention has been paid to whether NLRP3 can also regulate the malignant processes against drug resistance in cancer. For instance, the activated NLRP3 inflammasome facilitated leukemia cell proliferation and improved chemotherapy resistance, while the inactivation of NLRP3 exerted the opposite effects, demonstrating the promotive effects of NLRP3 on cancer development and chemotherapy resistance in acute myeloid leukemia [21]. Meanwhile, NLRP3 was reported to enhance gemcitabine-based resistance in triple-negative breast cancer cells [22]. In addition, it was proved that NLRP3 inflammasome could promote resistance of 5-fluorouracil to oral squamous cell carcinoma [23]. The existing evidence suggested that NLRP3 inflammasome might be an effective target for the adjuvant chemotherapy of multiple types of cancer. Regarding ovarian cancer, it was only reported by Alrashed et al. that NLRP3 could improve the gemcitabine sensitivity in gemcitabine-resistant ovarian cancer cell lines [20], but whether NLRP3 inflammasome also exerted critical effects on DDP resistance in ovarian cancer remained unclear. Here, a high level of NLRP3 was found in DPP-resistant ovarian tumors, whereas NLRP3 silencing can suppress cell proliferation, invasion, and migration and promote apoptosis of SKOV3/DPP and A2780/DPP cells. The abovementioned findings suggest that downregulation of NLRP3 repressed the malignant processes of DPP-resistant ovarian cancer cells, which might be beneficial to improve the antitumor effects of DDP against DDP-resistant patients with ovarian cancer.

EMT, an early event of tumor invasion and metastasis [19], is a fundamental developmental process wherein epithelial cells lose their polarity and gain invasiveness, eventually leading to the transformation into mesenchymal cells, which has been recognized as a major approach to propagating tumor dissemination. Therefore, along with this condition, the classical epithelial marker E-cadherin was downregulated, while mesenchymal markers, including fibronectin, vimentin, and N-cadherin, were upregulated [24–26]. Currently, EMT is considered a crucial indicator for not only cancer development but also drug resistance. Resistance to DDP-based chemotherapy in ovarian cancer was linked to EMT [27]. Blocking PI3K/Akt/mTOR signaling pathway attenuates ovarian cancer chemoresistance to DDP through reversing EMT [28]. Consistently, we also observed a restoration of EMT following NLRP3 silencing in DDP-resistant ovarian cancer cells in this study, as proved by the elevated level of E-cadherin and the reduced level of N-cadherin, vimentin, and fibronectin, suggesting that NLRP3 knockdown might alleviate resistance towards ovarian cancer via reversing EMT.

However, some limitations still exist in the present study. First, *in vivo* assay may be beneficial to validate the current findings *in vitro*. Secondly, this study preliminarily explored the molecular mechanism of NLRP3 in DDP-resistant ovarian cancer, and the in-depth research focusing on its potential mechanism is deserved to be conducted in our future work.

## 5. Conclusion

Taken together, the current research highlights the specific role of NLRP3 in DDP-resistant ovarian cancer. Silencing of NLRP3 can weaken drug resistance through repressing cell proliferation, invasion, and EMT in DDP-resistant ovarian cancer cells. NLRP3 is suggested to be a target for monitoring DDP resistance in ovarian cancer and improving therapeutic outcomes.

## Figures and Tables

**Table 1:** Clinical characteristics of ovarian carcinoma patients.

Pathological parameters	Nonresistant	DDP-resistant
Age		
<50	7	9
≥50	11	9
Stage		
I+II	13	9
III+IV	5	9
Lymph node metastasis		
Negative	17	17
Positive	1	1
Histologic subtype		
Serous	7	14
Others	11	4

**Table 2:** Primer of the target gene for real-time PCR.

Genes		Sequences (5′-3′)
NLRP3	Forward	5′-CTCTAGCTGTTCCTGAGGCTG-3′
	Reverse	5′-TTAGGCTTCGGTCCACACAG-3′
*β*-Actin	Forward	5′-AGCGAGCATCCCCCAAAGTT-3′
	Reverse	5′-GGGCACGAAGGCTCATCATT-3′

## Data Availability

All data can be obtained from the corresponding author on reasonable request.

## Abbreviations

DDP: Cisplatin
NLRP3: Nod-like receptor protein 3
qRT-PCR: Quantitative real-time PCR
EMT: Epithelial-mesenchymal transition
IL-18: Interleukin-18
DAB: Diaminobenzidine
PVDF: Polyvinylidene difluoride
TBST: Tris-buffered saline and Tween-20
sh-NLRP3: Short hairpin RNA targeting NLRP3
sh-vector: Empty vector
CCK-8: Cell counting kit-8
OD: Optical density
TUNEL: Terminal deoxynucleotidyl transferase-mediated dUTP nick end labeling
DAPI: 4′,6-Diamidino-2-phenylindole
SD: Standard deviation
ANOVA: One-way analysis of variance.

## Supplementary Materials

Supplementary Materials Scheme 1. Inhibition of NLRP3 enhances the susceptibility of cisplatin-resistant ovarian cancer cells to cisplatin treatment. NLRP3 was overexpressed in DDP-resistant ovarian cancer and activated the assembly of NLRP3 inflammasome. NLRP3 inflammasome could promote cell proliferation, invasion, and metastasis of DDP-resistant ovarian cancer and inhibit apoptosis of DDP-resistant ovarian cancer.
