# Supplementary figures and images for: Silencing of NLRP3 Sensitizes Chemoresistant Ovarian Cancer Cells to Cisplatin
Source: Mediators Inflamm. 2023 Jun 2;2023:7700673. doi: 10.1155/2023/7700673 (PMC10256449; doi:10.1155/2023/7700673)

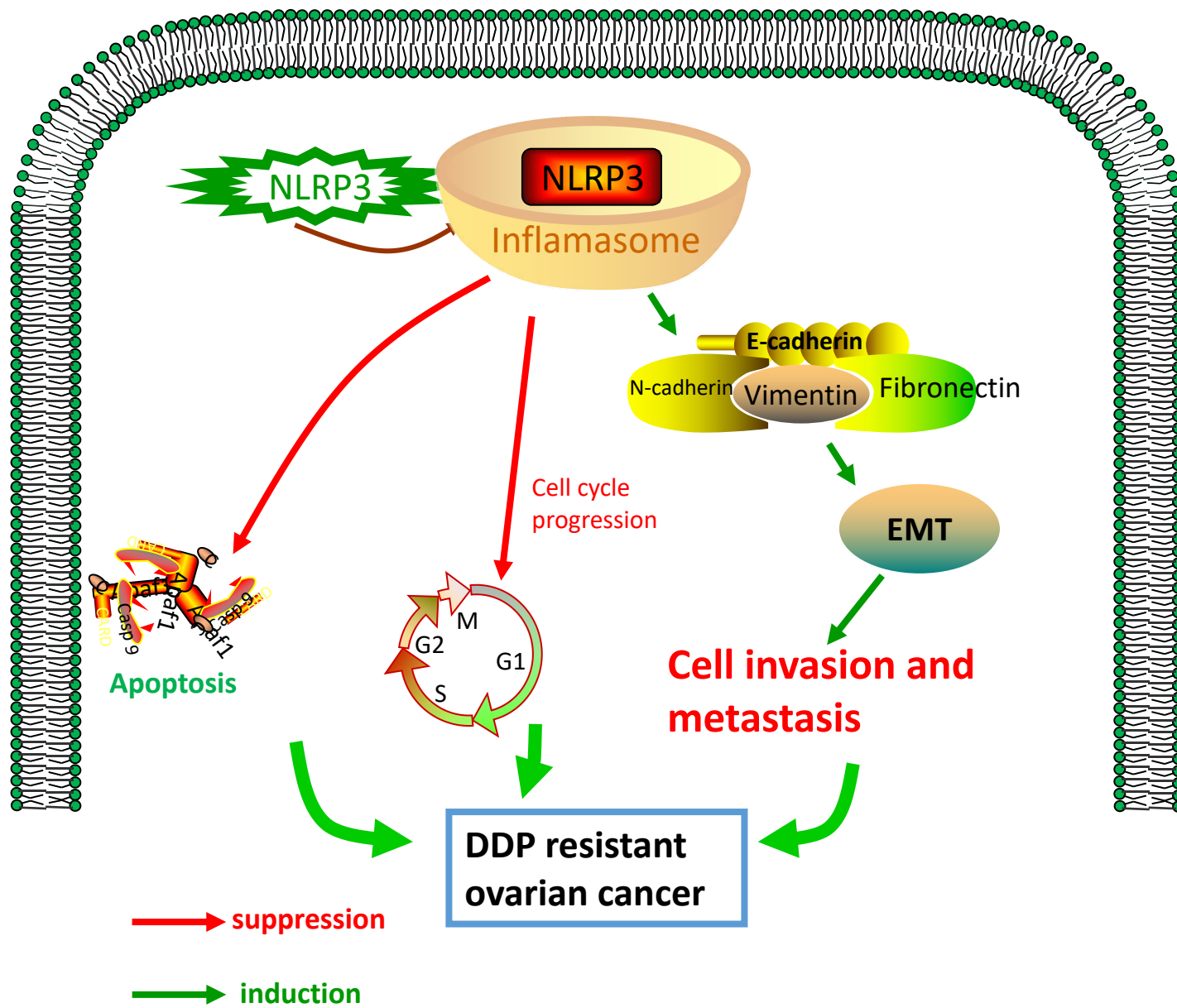

Supplement: Supplementary Materials — Scheme 1. Inhibition of NLRP3 enhances the susceptibility of cisplatin-resistant ovarian cancer cells to cisplatin treatment. NLRP3 was overexpressed in DDP-resistant ovarian cancer and activated the assembly of NLRP3 inflammasome. NLRP3 inflammasome could promote cell proliferation, invasion, and metastasis of DDP-resistant ovarian cancer and inhibit apoptosis of DDP-resistant ovarian cancer. [file 7700673.f1.pdf]
